# Bioaccumulation of Potential Harmful Elements in Fossorial Water Voles Inhabiting Non-Polluted Crops

**DOI:** 10.3390/toxics13121083

**Published:** 2025-12-16

**Authors:** Aitor Somoano, Roser Adalid, Jacint Ventura, Francesc Muñoz-Muñoz, Màrius Vicent Fuentes, Mario Menéndez-Miranda, Marcos Miñarro

**Affiliations:** 1 Servicio Regional de Investigación y Desarrollo Agroalimentario (SERIDA), Carretera AS-267, PK 19, 33300 Villaviciosa, Spain; 2 Secció Departamental de Parasitologia, Departament de Biologia, Sanitat i Medí Ambient, Universitat de Barcelona, 08007 Barcelona, Spain; 3 Departament de Biologia Animal, Biologia Vegetal i Ecologia, Universitat Autònoma de Barcelona, Bellaterra, 08193 Barcelona, Spain; 4 Natural Sciences Museum of Granollers, 08401 Granollers, Spain; 5 Parasites and Health Research Group, Department of Pharmacy, Pharmaceutical Technology and Parasitology, University of Valencia, 46100 Valencia, Spain

**Keywords:** *Arvicola scherman*, body condition, non-polluted habitats, cadmium (Cd), lead (Pb), mercury (Hg), selenium (Se)

## Abstract

Although the health risks associated with exposure to potentially harmful elements (PHEs) are well documented, there is still limited research on their accumulation at trace concentrations in small mammals inhabiting agricultural ecosystems. This study provides the first comprehensive assessment of PHE accumulation in fossorial water voles (*Arvicola scherman*) from two low-input apple orchards (Nava and Oles) located in Asturias, northwestern Spain, demonstrating its value as a potential bioindicator of trace element inputs. We quantified the concentrations of three toxic metals (Pb, Cd, and Hg) and selenium (Se), an element with concentration-dependent toxicity, in kidney, liver, and muscle tissues. We also determined inter-population differences and associations with body condition. Overall, element concentrations generally reflected the natural content of the local soils, except for Cd in the kidney, which exceeded soil levels, highlighting its strong affinity for this organ. Significant differences in Pb, Cd, and Se accumulation were found among tissues, with the kidney showing the highest levels, underscoring the importance of organ-specific monitoring. The observed positive correlations between body condition and Se and Cd in kidney tissue, and Cd in liver tissue, particularly in the Nava population, suggest that individual health status modulates exposure and accumulation dynamics. Higher PHE burdens were found in Oles specimens, pointing to a potential threshold effect where higher contamination may begin to impair physiological condition. In contrast, Hg showed a negative relationship with body condition, suggesting possible adverse effects even in these low-input systems. These findings highlight the importance of carefully interpreting physiological biomarkers within an ecological context and demonstrate the potential for trace elements to propagate through the food web, with possible cascading effects on predator health and key ecosystem services, such as natural pest control. Future research should extend to more contaminated sites and adopt an integrative framework combining biomonitoring, dietary ecology, and stress physiology to better assess the ecological risks posed by trace elements in agroecosystems.

## 1. Introduction

Pollution in ecosystems is exacerbated by anthropogenic activities such as mining, smelting, transportation, and industrial manufacturing, which release certain potentially harmful elements (PHEs) into the environment via atmospheric deposition, surface water, and soil [1]. Soil serves as a major sink for these elements [2,3], which can persist in ecosystems for extended periods [4]. In agricultural systems, practices like the use of metal-containing pesticides and fertilizers further elevate concentrations of some PHEs in surface horizons [1]. Although typically in lower concentrations, lithogenic metals can also mobilize and become bioavailable under certain conditions [1]. Non-essential metals such as arsenic (As), cadmium (Cd), and lead (Pb) accumulate in microorganisms, plants, and animal tissues over time [5,6]. These elements undergo biomagnification through trophic transfer, resulting in significant ecological and health consequences [7]. Health effects include mutagenesis, carcinogenesis, teratogenesis, immunosuppression, inhibited growth, and reduced fertility [8].

Small mammal species inhabiting polluted areas are likely to accumulate high concentrations of some PHEs [9,10,11]. In contrast, data on their uptake in micromammals from non-polluted habitats are scarce [12,13]. Most studies in such environments have focused on bioaccumulation in large mammals intended for human diet [14,15]. Although predatory small mammals that feed on soil-dwelling invertebrates tend to exhibit higher metal accumulation [16,17,18], phytophagous species, particularly those consuming hypogeal plant tissues, may also accumulate significant element concentrations due to elevated content in these tissues [3,19]. Despite their ecological significance as key prey species in agroecosystems [20,21], research on bioaccumulation in small herbivorous mammals is limited [22]. This knowledge gap is critical, given the cascading toxic effects that may propagate through food webs [23].

The montane water vole, *Arvicola scherman* (also considered as a fossorial form of *A. amphibius* by authors; see [24]), inhabits extensive burrow systems in grasslands and fruit orchards and primarily feeds on roots, bulbs, and tubers of dicotyledons and some Poaceae [25,26]. This rodent is found in mountainous regions across Europe, including the northern Iberian Peninsula, the Alps, central European mountains, and the Carpathians [27]. It is considered one of the most significant pest voles in European agroecosystems [25,28,29]. With a high reproductive potential [30], *A. scherman* populations can peak at densities of up to 1000 voles/ha [28], making it a critical prey species for numerous raptors and predatory mammals within these ecosystems [21,31]. Despite substantial literature on its ecology and population dynamics, no data is currently available on levels of PHE accumulation or their possible detrimental effects on fossorial water vole populations.

There is no clear consensus regarding the primary target organ for PHE accumulation in small mammals [17,22]. However, Cd, Pb, and Hg can be expected to exhibit organ-specific kinetics: Cd and Pb primarily accumulate in the kidney and liver, often bound to metallothioneins [32,33], while Hg is retained in muscle and other tissues depending on its form [34]. Co-exposure can exacerbate toxicity, particularly in renal tissues, via oxidative stress and disruption of antioxidant defenses [35]. Selenium may mitigate these effects by forming inert complexes and supporting antioxidant systems [17,36,37]. These kinetics and interactions are central to understanding tissue-specific accumulation and toxicity in small mammals.

We hypothesize that PHE concentrations could be higher in voles inhabiting crops influenced by historical agricultural practices and that metal uptake is further modulated by individual physiological state [9,10,16,38], with voles in better condition potentially ingesting or retaining greater amounts of these elements [30]. Studying trace-element accumulation in vole populations from well-characterized, low-contamination habitats [30,39,40] provides an unbiased framework to advance rodent biomonitoring. In this study we investigate the accumulation of three toxic metals (Pb, Cd, and mercury [Hg]) and one non-metal/metalloid (selenium [Se], whose toxicity depends on its concentration) in two populations of *A. scherman* inhabiting low-input apple orchards, a crop frequently damaged by this vole species in western Europe [41,42]. The specific objectives were (1) to evaluate the variability of these PHEs’ accumulation across liver, kidney, and muscle tissues; (2) to identify potential inter-population differences in concentrations; and (3) to investigate how the physiological state of voles, assessed through their body condition, influences the accumulation of these elements in their tissues. The findings could contribute to improved environmental health policies and integrated pest management strategies for this key vole pest.

## 2. Materials and Methods

### 2.1. Study Area

**Figure 1:**
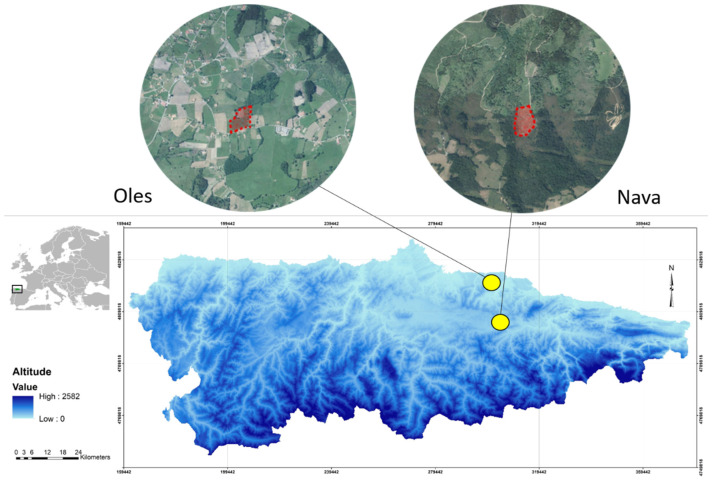
Location of Asturias (NW Spain) and the study apple orchards (Oles and Nava) in this region, indicated by yellow points. Orthophotos show agricultural area surrounding each plot.

The study was conducted in Asturias (Figure 1), a coastal region in northwestern Spain characterized by a temperate oceanic climate with mild temperatures and annual rainfall exceeding 1000 mm, evenly distributed throughout the year. The agroecosystem features a highly variegated bocage landscape (Figure 1), comprising a fine-grained mosaic of orchards, hay meadows, pastures, annual crops, eucalyptus plantations, settlements, and semi-natural woody vegetation patches, including temperate broad-leaved forests, heathland, and riverine forests [40].

Specimens were collected from two semi-intensive, low-input apple orchards, in the localities of Oles (43°31′48” N, 5°27′05” W) and Nava (43°22′25” N, 5°25′04” W). These two sites were selected based on prior evidence demonstrating that the fossorial water vole populations in Oles and Nava represent distinct and genetically isolated populations [40]. Both orchards are apple cultivar collections with 426 cultivars each planted on semi-dwarfing M7 rootstock, with spacing of 2.5–3 m between adjacent trees and 5.5–6 m between rows. The orchards maintained dense, evergreen grass cover year-round, with periodic shredding to avoid weed competition. Management adhered to organic guidelines, avoiding herbicides, synthetic pesticides and fertilizers [43]. Pest control included punctual applications of white oil, azadirachtin, and codling moth granulosis virus, while fungal diseases were managed with occasional copper and lime sulfur treatments. Organic fertilization was performed with vermicompost and no irrigation was used. The Oles orchard, established in 2004, spans 2.07 ha at 170 m a.s.l. and is surrounded by small plots of pastures, mowing meadows, orchards, corn crops, and eucalyptus plantations, typically separated by hedgerows or roads. The Nava orchard, established in 2002, covers 2.84 ha at 271 m a.s.l. and is bordered primarily by shrubland and broad-leaved forests. Previously, this site consisted of dense scrub.

Based on geostatistical analyses of geogenic trace elements in Asturias [44], the soils of both sampling areas are considered uncontaminated agricultural soils with respect to Pb, Cd, and Hg [45]. These concentrations may therefore serve as baseline values for agricultural soils not influenced by specific point-source contamination, in line with the requirements of Spanish Royal Decree 9/2005 [46]. Under this regulation, soils exceeding heavy-metal concentrations by more than 100 times their baseline values are classified as contaminated and must undergo further investigation through risk analysis.

### 2.2. Tissue Samples

A total of 70 adult specimens of *A. scherman* (body mass: 69–105 g) were collected between January and June 2012 during a population outbreak in the study area [36]. Sampling included 40 individuals from Oles and 30 from Nava, with an equal sex ratio recorded at both localities. Given their status as significant agricultural pests in Spanish grasslands and orchards [29], their population control is mandated under Spanish Royal Decree 409/2008 [47]. Sustainable population control was conducted using snap traps (Topcat^®^, Andermatt Biocontrol, Grossdietwil, Switzerland) placed in burrows, checked twice daily over a maximum of five days. This trapping method is equally effective in capturing individuals regardless of their sex, age, or physiological state [39]. Shortly after capture specimens were cryopreserved at −20 °C until necropsy. Specimens were sexed and aged according to criteria established by Somoano et al. [39]. Head and body length and body mass were recorded for each individual. Specimens belonged to age classes IV (14–30 weeks) and V (older than 30 weeks) as defined by Somoano et al. [39]. Dissections were performed with sterilized stainless-steel instruments, and tissue samples (kidney, liver, and muscle) were collected, stored in glass vials, and maintained at −20 °C until processing for trace element analysis. This study was classified under zootechnical purposes, exempting it from ethical approval requirements [48]. All procedures complied with European Directive 2010/63/EU [49] on the protection of animals used in scientific research.

### 2.3. Chemical Analysis

Tissue samples (~150 mg wet weight) were digested in Teflon vessels using 2 mL of HNO_3_ and 1 mL of H_2_O_2_ (Merck, Suprapure, Merck KGaA, Darmstadt, Germany) at 90 °C overnight. All materials used during digestion were thoroughly acid-rinsed to prevent contamination. After digestion, samples were diluted with 30 mL of Milli-Q water and analyzed for Cd, Pb, Se and total Hg using inductively coupled plasma mass spectrometry (ICP-MS, Perkin Elmer Elan 6000, PerkinElmer, Waltham, MA, USA). The analytical procedure was validated with standard reference materials: dogfish (*Squalus acanthias*) liver (DOLT-3) and muscle (DORM-2) from the National Research Council of Canada. All measurements from the certified reference materials fall within a range of 10% of the certified value for the four elements analyzed, thus validating the methodology. Several analytical blanks, analyzed following the same procedure as the samples, were included to determine detection limits (0.11, 0.05, 0.16, 0,13 ppb for Pb, Cd, Se and Hg, respectively). All analyses were conducted at the CCiTUB (Scientific and Technological Centers of the University of Barcelona). Concentrations of total trace elements are expressed as mean (± SD) ng g^−1^ wet weight.

### 2.4. Statistical Analysis

Body condition index (BCI) was assessed using the approach described by Peig and Green [50], which relies on the standardized major axis (SMA) regression. The Scaled Mass Index (SMI) adjusts the body mass of an individual to a fixed value of a linear body measurement, reflecting the allometric relationship between mass and length. The calculation follows the formula: *SMI* = *m_i_*(*L*_0_/*L_i_*)*^bSMA^* where *m_i_* and *L_i_* are the body mass and the linear head and body length of individual *i*, respectively, *L*_0_ is a fixed reference value of *L* (arithmetic mean of head and body length for the study population), and *b_SMA_* is the scaling exponent derived from the SMA regression of ln(mass) against ln(length). Specimens of *A. scherman* previously analyzed in Somoano et al. [39] were used as reference population data. This method allows for standardization of body mass based on structural size, improving comparability across individuals with differing body proportions [10,50]. The specimens were collected from a population that appeared healthy, with ample food resources and no evident signs of environmental stress. Consequently, body condition scores are expected to reflect physiological status rather than being confounded by nutritional deficiencies. In addition, the BCI estimates were derived from a considerably larger dataset of individuals from the same populations [39], thereby reducing the likelihood of sampling bias.

Prior to site comparisons, data from each tissue were tested for homogeneity of variances (Levene’s test) and normality (Shapiro–Wilk test). Normality tests revealed that 8 out of 12 variables deviated from normal distribution, requiring the application of nonparametric statistical methods. Sexual differences in trace element concentrations were assessed using the Mann–Whitney U test, but because significant differences were only observed in one case (Hg levels in kidney, U = 235, *p* = 0.002), data from both sexes were pooled for subsequent analyses. Relationships between BCI and trace element concentrations in each organ were evaluated using Spearman correlations. Differences in concentrations among tissues and between sites were analyzed with Kruskal–Wallis and Mann–Whitney U tests, respectively. Statistical significance was established at *p* < 0.05. To minimize the risk of type I errors in multiple pairwise comparisons, a Bonferroni correction (α = 0.05/number of comparisons) was applied [51]. Principal component analysis (PCA) was conducted separately for each tissue to identify patterns of variation in concentrations and relationships among variables. This tissue-specific approach accounts for differences in metal accumulation and variability among organs, providing a clearer and biologically meaningful framework for interpreting trace element data in ecotoxicological studies [52,53]. All statistical analyses were performed using Statistica 8.0 (StatSoft Inc. 2007, Tulsa, OK, USA) and Python Language Reference (Version 3.11, Python Software Foundation, Wilmington, DE, USA).

## 3. Results

**Figure 2:**
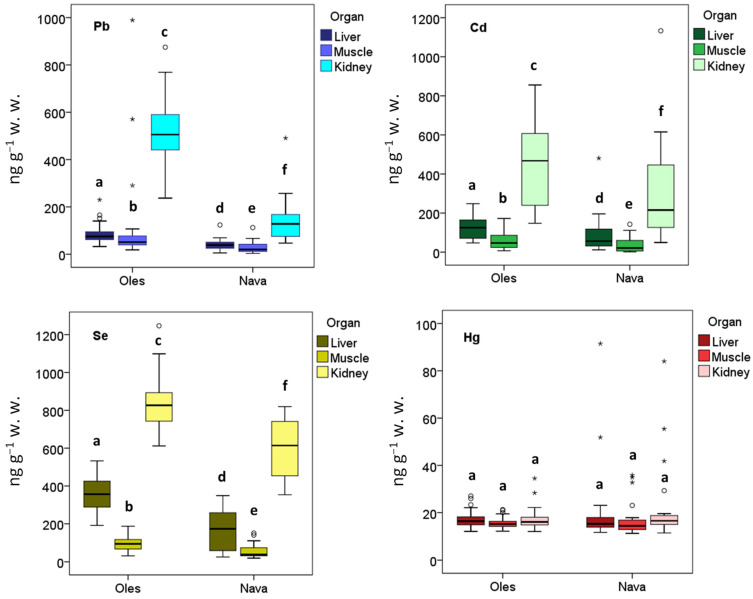
Values of the metal concentrations (ng g^−1^ w. w.) obtained in the three analyzed tissues of *A. scherman* in each of the two sampling sites (Nava and Oles). The dark line in the middle of the boxes shows the median, the bottom and the top of the box indicate the 25th and 75th percentile, respectively, and the T-bars that extend from the boxes mark the minimum and maximum values. Circles indicate outliers (values lying between 1.5 and 3 times the interquartile range from the quartiles), whereas asterisks indicate extreme outliers (values lying more than 3 times the interquartile range). For each sampling site and heavy metal, boxplots with the same letter are not significantly different.

Concentrations of PHEs varied significantly among tissues, regardless of the population (Pb: H’ = 112.09, *p* < 0.01; Cd: H’ = 130.69, *p* < 0.01; Se: H’ = 166.26, *p* < 0.01; Hg: H’ = 10.63, *p* < 0.01). In specimens from Nava, Pb, Cd, and Se levels were significantly higher in the kidney, followed by the liver and muscle (*p* < 0.001) (Table 1, Figure 2). A similar trend was observed in specimens from Oles, except for Pb, which was higher in the kidney, followed by muscle and liver (*p* < 0.001) (Table 1, Figure 2). Mercury (Hg) concentrations showed no significant differences among tissues in either population (*p* > 0.01).

When comparing sites (Table 1, Figure 2), Pb, Cd, and Se concentrations were significantly higher in Oles across all tissues (*p* < 0.001). Specifically, average Pb, Cd, and Se levels in liver, kidney, and muscle were, respectively: 2.2, 1.5, and 2.1 times; 3.7, 1.5, and 1.4 times; and 3.6, 1.4, and 1.6 times higher in Oles than in Nava. No significant site differences were observed for Hg concentrations.

According to the applicable food-safety legislation, which sets maximum levels for certain contaminants in foodstuffs [54], most Cd and Pb concentrations complied with the established limits, with only two exceedances observed. Muscle Cd at Oles slightly exceeded the regulatory threshold (53.1 vs. 50 ng g^−1^ w. w.), while renal Pb at the same site displayed a pronounced exceedance (518 vs. 200 ng g^−1^ w. w.). Muscle Pb at Oles approached (Table 1), but did not surpass, its corresponding limit (100 ng g^−1^ w. w.). All remaining Cd and Pb concentrations in liver, kidney, and muscle from both sites were well below the regulatory maximum levels [54].

**Figure 3:**
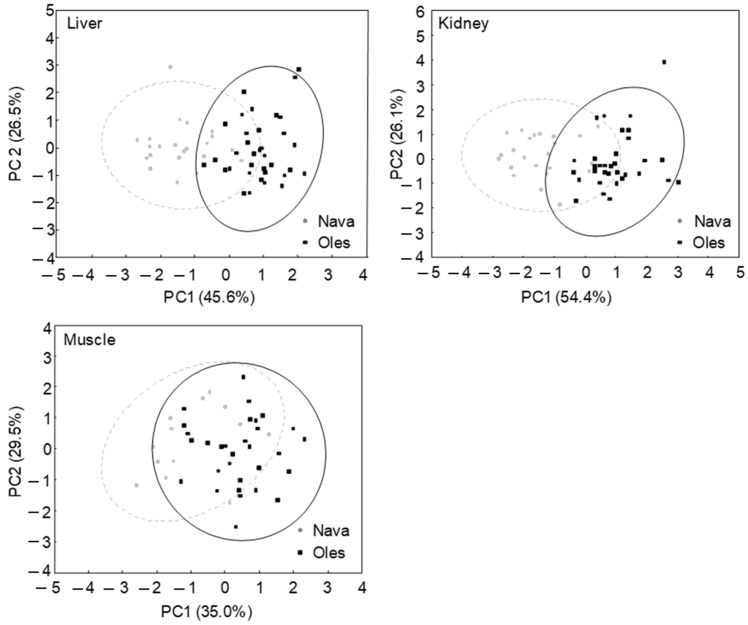
Distribution of the specimens of *A. scherman* from Oles and Nava (NW Spain) in the space of the two first principal components (PC1 and PC2) in the principal component analyses corresponding to each tissue analyzed (kidney, liver, and muscle). Confidence ellipses indicate the 95% multivariate confidence regions for each population, based on the dispersion of the specimens in the PC1–PC2 space, and illustrate the degree of variability and overlap between populations.

Principal component analysis (PCA) revealed that the first two principal components (PC1 and PC2) explained a substantial proportion of the variance in PHEs concentrations: 72.1% for the liver, 80.5% for the kidney, and 64.5% for the muscle (Table 2, Figure 3). In liver and kidney tissues, Pb, Se, and Cd contributed strongly to PC1, while Hg was closely associated with PC2. In muscle tissue, Cd and Hg were tightly associated with PC1, whereas Se and Hg were more strongly linked to PC2 (Table 2, Figure 3).

**Figure 4:**
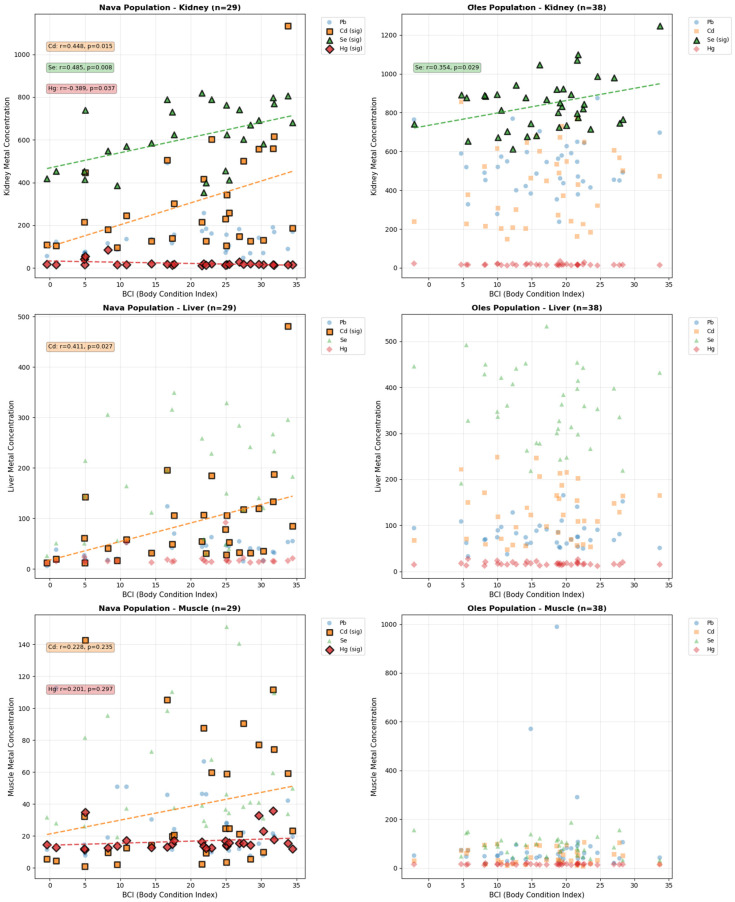
Correlations between body condition index (BCI) and Pb, Cd, Se and Hg concentrations (ng g^−1^ w. w.) in each studied organ from *A. scherman* specimens captured in the two sampling sites (Nava and Oles). Cases in which the correlations were statistically significant are highlighted in bold, and the corresponding regression lines are shown as dashed lines.

In the Nava population, BCI correlated positively with Se (r_s_ = 0.485, *p* = 0.008) and Cd (r_s_ = 0.448, *p* = 0.015) concentrations in kidney, as well as for Cd in liver (r_s_ = 0.411, *p* = 0.027) (Figure 4). Additionally, a negative correlation was found between BCI and Hg in the kidney (r_s_ = −0.389, *p* = 0.037). In contrast, the Oles population exhibited fewer and weaker associations of BCI with element concentrations, with only a positive correlation between BCI and Se in kidney (r_s_ = 0.354, *p* = 0.029) reaching statistical significance.

## 4. Discussion

In this study we provide the first detailed analysis of the accumulation of PHEs in the tissues of fossorial water voles inhabiting agricultural habitats. The concentrations of Pb, Cd, Hg, and Se measured in kidney, liver, and muscle tissues are consistent with those reported for surrounding soils [44], which correspond to uncontaminated agricultural plots [45]. These findings suggest that the natural trace element content of the soils, largely determined by the parent rock material [13,44], is a key factor influencing bioaccumulation in local fauna. The PHE concentrations measured in fossorial water voles in 2012 may serve as a preliminary baseline for evaluating the current and future health status of their populations. Moreover, the broad distribution of this species across agricultural landscapes in northern Spain [41] facilitates PHE monitoring in individuals inhabiting contaminated croplands, including areas where elevated pollutant levels have recently been reported [55].

In the locality of Oles, however, higher levels of Pb, Cd, and Se in vole tissues compared to those from Nava may suggest the presence of additional PHE inputs. Although no direct environmental measurements are available, plausible contributors could include agricultural practices, such as the historical use of certain pesticides and fertilizers [2,56], as well as past small-game hunting activities that might have introduced lead into the environment [57,58]. These inter-population differences highlight the sensitivity of *A. scherman* as a potential bioindicator of spatial variation in environmental trace element exposure. PHE concentrations in tissues likely reflect not only environmental exposure but also differences in dietary composition and foraging behavior [10], including potential preferences for plant species that accumulate trace elements. This interpretation aligns with the known feeding habits of fossorial water voles, which consume a wide range of plants with varying capacities to bioaccumulate metals. Preferred dietary items such as apple trees (*Malus domestica*) [59] and dandelions (*Taraxacum officinale*) [60], as well as other common plants in the study area, including bindweed (*Convolvulus arvensis*), alfalfa (*Medicago* spp.), and shepherd’s purse (*Capsella bursa-pastoris*) [36,61,62], are all known metal accumulators. These observations underscore the multifactorial and context-dependent dynamics of trace element accumulation in wild small mammals, reinforcing the need to interpret bioaccumulation data within a broader ecological framework to avoid misattributing natural variability to pollution effects.

The kidney exhibited the highest capacity for accumulation, followed by the liver and muscle. This pattern is consistent with findings for other small mammals, such as the short-tailed vole (*Microtus agrestis*) in polluted agroecosystems [17]. Notably, Cd levels in the kidney exceeded those measured in soil, a physiologically plausible pattern given the strong renal retention of this metal. Cadmium induces metallothioneins, forming Cd–MT complexes that are filtered by the glomerulus and reabsorbed in proximal tubules, leading to persistent renal accumulation. Similar patterns in small mammals have been documented by Fritsch et al. [32] and Elturki [33], supporting the suitability of the kidney as a target organ for monitoring Cd bioaccumulation. In contrast, mercury concentrations were low across all tissues and populations, with no significant organ-specific accumulation. The PCA results support this observation, indicating that Hg was a secondary factor in explaining tissue contamination. The higher Pb, Cd, and Se levels in Oles compared to Nava, combined with the observed organ-specific accumulation trends, provide valuable insights on trace element bioavailability.

Body condition is a key parameter in ecotoxicological biomonitoring [17,37], but its interpretation must account for factors such as age, reproductive status, and habitat [10]. After controlling for these variables, the analysis reveals population-specific patterns in the relationships between PHE concentrations and BCI. Overall, the Nava population appears to show somewhat stronger associations between BCI and PHE concentrations, particularly for Se and Cd in kidney tissue. This may tentatively indicate that individuals in better body condition could accumulate slightly higher levels of these elements. This aligns with Tête et al. [10], who reported a threshold-dependent relationship in *Apodemus sylvaticus*, with Cd increasing alongside body condition in moderately polluted areas without major physiological effects. Since body condition reflects energy reserves and nutritional status [30], healthier individuals may ingest or retain more PHEs in slightly contaminated environments, whereas negative relationships between body condition and Cd or Pb are observed in heavily polluted sites [10]. In the present study, Hg in the kidney showed a negative correlation with BCI, suggesting adverse physiological effects even in habitats considered non-polluted. Given that Cd, Pb, and Hg can potentiate each other’s toxicity [35], particularly in renal tissues, this association may reflect the combined burden of these metals rather than the action of Hg alone [63]. At the same time, the presence of Se, which can attenuate the toxicity of Hg [64] and, to a lesser extent, Cd [65] and Pb [66] by forming inert complexes and supporting antioxidant defenses, may partially buffer these effects and influence the strength of the observed correlations. Although Oles voles had higher PHE burdens, they showed weaker BCI–PHE associations, likely due to interactions between enhanced Cd, Pb, and Hg effects and the concurrent modulatory role of Se [64,65,66]. Only renal Se displayed a positive correlation with BCI, consistent with its protective function. Finally, the disappearance of the positive Cd–BCI relationship at higher Cd levels suggests that any initial compensatory response is lost once toxicity surpasses a threshold, potentially reinforced by synergistic effects with Pb and Hg [63].

During population outbreaks, fossorial water voles provide an exceptionally abundant and easily accessible biomass—reaching several tens of kg·ha^−1^ in grasslands—which is exploited by a diverse community of predators [31,67]. Among these, carrion crows, common buzzards, and red kites play a dominant role, supporting substantial portions of their daily energetic demands (147, 182 and 240 g·day^−1^ respectively), followed by terrestrial carnivores such as red foxes, domestic cats, and wildcats (480, 360, and 392 g·day^−1^ respectively) [31]. Smaller raptors and nocturnal predators, including hen harriers, kestrels, and long-eared owls, contribute to a lesser extent (116, 79, 84 g·day^−1^ respectively), while omnivorous mammals like badgers feed on voles opportunistically (786 g·day^−1^) [31]. This extraordinary prey availability facilitates the transfer of metal contaminants from voles through the food web, with potential cascading ecological effects [1,7,57]. Predators, which typically exhibit limited detoxification capacity, may bioaccumulate metals throughout their lifespans [22], thereby heightening their vulnerability to reproductive impairment and physiological deterioration [8,68]. The slight exceedance of Cd in muscle and the pronounced above-limit concentrations of Pb in kidneys observed in fossorial water voles from Oles [54] align with this vulnerability, suggesting that chronic exposure may already be contributing to elevated body burdens in predators. Such toxic effects could reduce predator efficiency and alter their role in natural pest control [69,70], threatening the provision of an essential ecosystem service. To better assess environmental risks associated with trace elements in agricultural settings, future studies should focus on contaminated areas where *A. scherman* serves as a keystone prey species. Comprehensive research integrating biomarkers, histological analyses, diet assessments, and environmental variables is needed to disentangle the multiple stressors affecting wildlife health. This integrative approach, aligned with the emerging field of stress ecology, could advance our understanding of pollutant effects under real-world conditions and inform science-based strategies to preserve ecological balance in agroecosystems.

## 5. Conclusions

This study provides the first comprehensive assessment of potentially harmful element (PHE) accumulation in fossorial water voles (*A. scherman*) inhabiting agricultural habitats, demonstrating this species is a potential bioindicator of trace element inputs. Organ-specific accumulation, especially the high Cd and Se levels in kidney tissue, underscores the relevance of tissue selection in monitoring programs. The observed associations between body condition and PHE concentrations indicate that individual health status and dietary habits modulate exposure and accumulation dynamics but also reveal potential threshold effects where higher contamination may negatively affect physiological condition. Concentrations of Hg showed a negative relationship with body condition, suggesting possible adverse effects even in non-polluted habitats. These findings emphasize the need for cautious interpretation of physiological biomarkers, considering ecological and biological variability. Given the key ecological role of *A. scherman* as prey for multiple predators, these findings stress the potential for metal transfer through the food web, with implications for predator health and ecosystem services such as natural pest control. Future research should expand into contaminated sites and adopt an integrative framework combining biomonitoring, dietary ecology, and stress physiology to better understand the ecological risks posed by trace elements in agroecosystems.

## Figures and Tables

**Table 1:** Mean (± SD), as well as minimum and maximum concentrations (ppb; ng g^−1^ w. w.), of potentially harmful elements in the three analyzed tissues at each of the two sampling sites, along with the results of the Mann–Whitney tests comparing metal concentrations between sites. Reference concentration ranges (minimum and maximum) for heavy metals in the soil of each sampling area are also provided (Domínguez-López, 2015 [46]).

		Nava					Oles					Mann–Whitney U Results	
Tissue	Metal	N	Mean	± SD	Min	Max	N	Mean	± SD	Min	Max	U	*p* Corrected
Liver	Pb	30	39.29	22.26	5.66	123.57	40	85.02	37.26	32.54	230.10	108.00	<0.0001
	Cd	30	87.91	91.84	11.55	480.74	40	130.03	58.27	47.16	248.02	299.00	<0.0001
	Se	30	172.82	100.94	25.35	349.11	40	353.83	85.43	191.40	532.63	110.00	<0.0001
	Hg	30	19.51	15.34	11.66	91.50	40	17.18	3.39	12.06	27.05	486.00	0.1760
Kidney	Pb	30	136.36	83.43	47.06	490.86	40	518.04	133.80	237.28	874.81	20.00	<0.0001
	Cd	30	297.58	234.57	49.40	1133.12	40	446.74	198.81	147.63	855.46	315.00	0.001
	Se	30	606.17	150.51	353.35	819.17	40	840.69	131.84	611.61	1246.61	142.00	<0.0001
	Hg	30	21.03	14.84	11.42	84.01	40	17.05	4.25	12.02	34.50	544.00	0.5060
Muscle	Pb	30	26.92	22.99	3.36	112.52	40	97.30	171.12	18.20	989.20	181.00	<0.0001
	Cd	30	37.24	39.38	0.98	142.56	40	53.06	36.79	7.03	172.40	395.00	0.015
	Se	30	55.95	35.53	19.29	150.99	40	93.39	38.91	31.72	186.89	254.00	<0.0001
	Hg	30	16.58	6.53	11.24	35.84	40	15.49	2.22	12.17	21.26	523.00	0.3610
Metal reference values	Pb				18,545.00	25,102.00				25,102.00	35,981.00		
	Cd				296.34	418.14				296.34	700.12		
	Hg				120.37	135.40				170.91	294.79		

**Table 2:** Results from the principal component analyses performed in the three tissues.

Tissue	PC	Eigenvalue	% Variation	Factor-Variable Correlations			
				Pb	Cd	Se	Hg
Liver	PC1	1.824	45.59	0.391	0.215	0.338	0.056
	PC2	1.060	26.51	0.055	0.150	0.146	0.649
	PC3	0.714	17.86	0.019	0.620	0.067	0.294
	PC4	0.402	10.04	0.535	0.015	0.448	0.002
Kidney	PC1	2.177	54.43	0.342	0.288	0.350	0.020
	PC2	1.044	26.09	0.013	0.036	0.079	0.872
	PC3	0.513	12.83	0.282	0.630	0.017	0.071
	PC4	0.266	6.65	0.362	0.046	0.554	0.037
Muscle	PC1	1.401	35.02	0.188	0.439	0.109	0.264
	PC2	1.179	29.47	0.012	0.026	0.593	0.369
	PC3	0.931	23.27	0.750	0.210	0.005	0.035
	PC4	0.489	12.24	0.050	0.325	0.293	0.332

## Data Availability

The data supporting this study are openly available in the Zenodo repository at https://doi.org/10.5281/zenodo.17650697 (accessed on 10 November 2025).
